# Curcumin-Functionalized Graphene Oxide Strongly Prevents *Candida parapsilosis* Adhesion and Biofilm Formation

**DOI:** 10.3390/ph16020275

**Published:** 2023-02-11

**Authors:** Margherita Cacaci, Damiano Squitieri, Valentina Palmieri, Riccardo Torelli, Giordano Perini, Michela Campolo, Maura Di Vito, Massimiliano Papi, Brunella Posteraro, Maurizio Sanguinetti, Francesca Bugli

**Affiliations:** 1 Dipartimento di Scienze Biotecnologiche di Base, Cliniche Intensivologiche e Perioperatorie, Università Cattolica del Sacro Cuore, 00168 Rome, Italy; 2 Dipartimento di Scienze di Laboratorio e Infettivologiche, Fondazione Policlinico Universitario A. Gemelli IRCCS, 00168 Rome, Italy; 3 Istituto dei Sistemi Complessi, Centro Nazionale Ricerche (CNR), 00185, Rome, Italy; 4 Dipartimento di Neuroscienze, Università Cattolica del Sacro Cuore, 00168 Rome, Italy; 5 Fondazione Policlinico Universitario “A. Gemelli” IRCSS, 00168, Rome, Italy; 6 Dipartimento di Scienze Mediche e Chirurgiche Addominali ed Endocrino Metaboliche, Fondazione Policlinico Universitario A. Gemelli IRCCS, 00168 Rome, Italy

**Keywords:** biofilm, biofilm-related infections, natural anti-biofilm compounds, graphene

## Abstract

*Candida parapsilosis* is the major non-*C. albicans* species involved in the colonization of central venous catheters, causing bloodstream infections. Biofilm formation on medical devices is considered one of the main causes of healthcare-associated infections and represents a global public health problem. In this context, the development of new nanomaterials that exhibit anti-adhesive and anti-biofilm properties for the coating of medical devices is crucial. In this work, we aimed to characterize the antimicrobial activity of two different coated-surfaces, graphene oxide (GO) and curcumin-graphene oxide (GO/CU) for the first time, against *C. parapsilosis*. We report the capacity of GO to bind and stabilize CU molecules, realizing a homogenous coated surface. We tested the anti-planktonic activity of GO and GO/CU by growth curve analysis and quantification of Reactive Oxigen Species( ROS) production. Then, we tested the antibiofilm activity by adhesion assay, crystal violet assay, and live and dead assay; moreover, the inhibition of the formation of a mature biofilm was investigated by a viability test and the use of specific dyes for the visualization of the cells and the extra-polymeric substances. Our data report that GO/CU has anti-planktonic, anti-adhesive, and anti-biofilm properties, showing a 72% cell viability reduction and a decrease of 85% in the secretion of extra-cellular substances (EPS) after 72 h of incubation. In conclusion, we show that the GO/CU conjugate is a promising material for the development of medical devices that are refractory to microbial colonization, thus leading to a decrease in the impact of biofilm-related infections.

## 1. Introduction

Biofilm-related infections represent a major clinical threat with high impact on economic costs and human health [1]. Biofilms are surface-associated microorganism communities, composed of single or multi species, embedded in an extracellular polymeric substance (EPS), secreted by the cells [2,3]. Biofilm formation takes place through specific steps that include early adhesion to the surface, the formation of cellular aggregates as microcolonies, and the subsequent production of the extracellular matrix, leading to the formation of a complex microbial stratification with a highly organized architecture that is defined as a mature biofilm. This complex organization of microbial growth evolves with the disintegration of the apical components of the biofilm and the detachment of planktonic cells that may colonize other sites [2,4,5]. The EPS is a key component of the biofilm; in fact, the EPS contributes to the absorption of nutrients and, thanks to its physio-chemical characteristics, contributes to the mechanism of tolerance towards the human immune system and antimicrobial therapies [6,7,8]. The EPS is mainly composed of water, polysaccharide, and proteins, and, to a lesser extent, extracellular DNA; the composition can vary according to the microbial species and the environment [9,10,11].

Biofilms can form on biotic and abiotic surfaces and are widespread in marine and terrestrial environments; moreover, biofilms can also colonize human tissues, such as the skin, the vagina, and the gastrointestinal tract [12,13,14]. Microbial biofilm can also cause infection in humans, starting from the colonization of abiotic surfaces such as intravascular catheters, urinary catheters, prosthetic joints, heart valve prostheses, implanted cardiac devices, vascular prosthesis, and endotracheal tubes [6,15]. These biofilm-related infections are difficult to threat, often requiring prolonged and combined antimicrobial therapy with poor therapeutic outcomes and a high risk of drug resistance onset. In particular, the colonization of medical devices often requires the removal and replacement of the same, leading to a prolongation of the patient’s hospitalization and an increased risk of mortality [1].

*Candida* spp. are currently considered a top cause of candidemia, particularly bloodstream infections that are highly associated with the formation of biofilms on central venous catheters (CVCs) [15,16]. In the past decades, although *C. albicans* remained the predominant etiology, accounting for 50% of all candidemia cases, an epidemiological shift has been observed, highlighting non-albicans *Candida* spp. and predominantly *C. parapsilosis* as the major causes of CVC-related candidemia [17,18].

In this context, the development of new nanomaterials to produce medical devices with antimicrobial and anti-adhesive properties appears crucial; research focuses on the study of nanomaterials that exhibit anti-adhesion and anti-biofilm features to decrease the rate of contaminated devices and, consequently, the rate of device-related infections [19]. Among carbon nanomaterials, graphene oxide (GO) shows extensive multifactorial properties: stretchability, electrical and high thermal conductivity, and a large surface area that allows functionalisation with antimicrobial compounds. Moreover, GO has antimicrobial properties that can vary depending on the GO sheets’ dimension and the environment [20,21]. Briefly, GO displays three different antimicrobial mechanisms: GO sharp edges can physically cut the membrane, with the consequent leakage of the intracellular contents, GO can also induce the production of ROS, and, finally, GO sheets can wrap around microorganisms, isolating them from the environment, limiting the adsorption of nutrients, and stopping the proliferation [22,23,24,25,26]. When GO is used as coating and is casted on a surface, the interaction between the GO edges and microorganisms is limited and consequently, the antimicrobial activity decreases [23]. To enhance the antimicrobial properties of the GO-coated surface, numerous compounds and nanoparticles, such as silver, iron, and titanium nanoparticles with proven antimicrobial activities, have been employed to functionalize GO-coated surfaces [22].

Among different antimicrobial compounds, curcumin (CU) is a natural lipophilic polyphenolic obtained from the rhizome of the *Curcuma longa* plant with anti-inflammatory, antioxidant, anticancer, and antidiabetic properties [27]. Curcumin’s mechanism of antimicrobial action has been extensively investigated [28,29]. Curcumin appears to be able to inhibit cell proliferation [29], to damage bacterial cell membranes and cell walls [30,31], to inhibit the bacterial SOS response, and to downregulate the expression of virulence genes [32]. Interestingly, CU displays anti-biofilm activities, being able to interfere with the biofilm formation process through the inhibition of the quorum sensing system [27,33,34]. However, contradictory results on CU efficacy against *Candida* spp. have been reported [35,36]. This is probably due to CU’s poor solubility in water and poor stability, which is highly dependent on pH [37], and the bioavailability is therefore very low [27]. The use of nanomaterials, such as GO, that incorporate CU may increase its stability and availability. Due to its hydrophobic nature, CU spontaneously adsorbs on the GO surface, without the need for chemical agents, favouring the formation of a green coating with antimicrobial characteristics. Indeed, our group has successfully demonstrated the possibility of creating GO/CU nanoparticles active against different types of pathogens [31,38]. Bugli et al. [31] previously performed cytotoxicity tests on GO/CU, concluding that this innovative compound shows cytocompatibility on 3T3 fibroblasts compared to GO. Indeed, curcumin functionalization seems to limit the mortality, oxidative, and hemolytic effects of GO. GO has hydrophobic graphitic patches in between oxygen functional groups. In our previous work, we discussed how the curcumin attaches on the GO surface thanks to the π–π binding, as previously demonstrated for other aromatic molecules, such as ginseng, vitamin C, melatonin, and polyphenols of green tea [31]. In this context, several research in the last years focused on the conjugation of CU with polymers, nanoparticles, and nanocarriers to increase its bioavailability [39,40,41,42,43]. In a recent paper published in 2021 [39], the authors describe the development of a nanocomposite film made of renewable castor oil-based PU (polyurethan) with curcumin-modified GO nanosheets for wound dressing. The data revealed a stable and biocompatible nanocomposite film with wound-healing properties. Rahman and collaborators describe the fabrication of a complex composed of curcumin and graphene oxide encapsulated in liposomes, showing an anti-bacterial effect against *S. aureus* along with long-term stability of the complex over three months. Another significant research area focused on the use of personal protective equipment (PPE) functionalized with polymeric material with antimicrobial action. This is even more critical if we consider the on-going COVID-19 pandemic and its consequences. A review by Chiari W. and collaborators focused on several studies regarding the use of nano-structured polymers for the fight against COVID-19 [44]. In this regard, nano-engineered cotton materials with important antimicrobial activities were obtained using a graphene surface functionalized with 4-aminosalicyclic acid. These antimicrobial tissues can be applied in different areas including healthcare settings to improve PPE [45]. In the same contest, curcumin-treated wool fabric with antimicrobial ability was generated by Han S. and Yang Y. [46].

This study aimed to assess the anti-adhesive and anti-biofilm properties of GO and GO/CU surfaces against a high biofilm-forming (HBF) strain of *C. parapsilosis* isolated from a CVC of a patient with candidemia and previously characterized as HBF [47]. Fourier transform infrared spectroscopy (FTIR) spectra demonstrated the efficient functionalization of the surfaces, and the antifungal activity of GO/CU in comparison with GO alone was analyzed by Colony Forming Units (CFU) counting, crystal violet quantification of the biofilm, growth curves, FDA, ROS assay, and live and dead staining. Taken together, our data reveal high antifungal efficacy in terms of early adhesion and toward the formation of a mature biofilm. Considering the already well established cytocompatibility, curcumin-loaded graphene oxide has the potential to be used in the field of medical device functionalization.

## 2. Results

### 2.1. Characterization of GO- and GO/CU-Coated Surfaces

**Figure 1:**
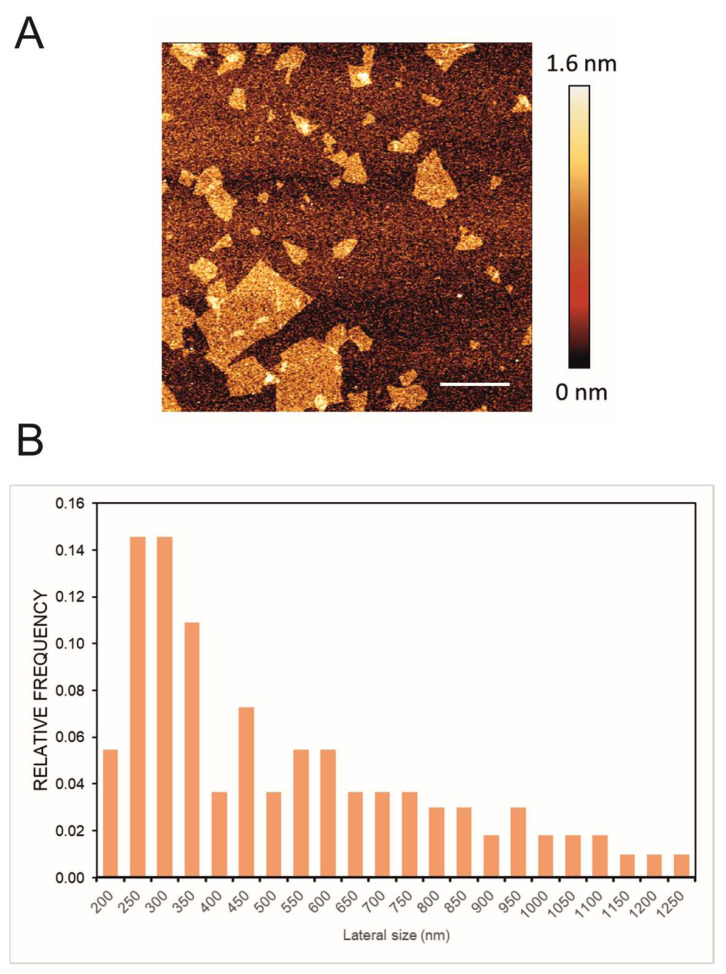
(**A**) Representative AFM image of GO; the scale bar is 1 µm. (**B**) Lateral size distribution of the flakes obtained through the analysis of three different images by ImageJ Fiji Software (Version 1.53 c).

**Figure 2:**
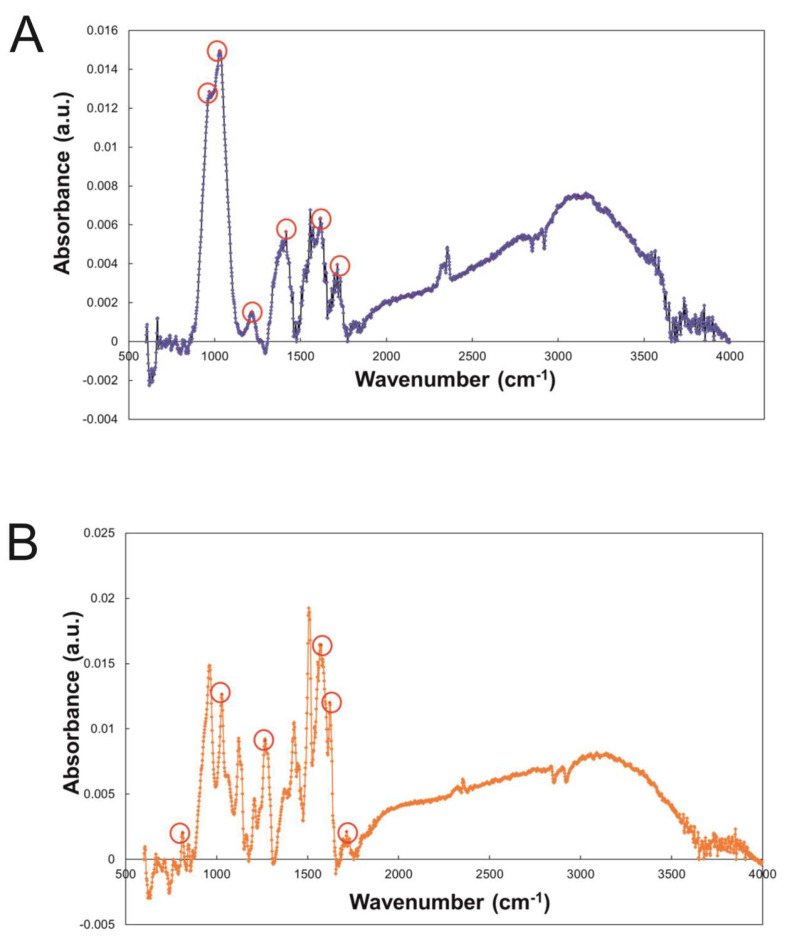
(**A**) FTIR spectroscopy of GO and (**B**) GO/CU with characteristic peaks highlighted. The material was directly laid on the ATR crystal, and the spectra were recorded.

In Figure 1A, a representative image of GO flakes is shown and GO had an average lateral size of 560 nm estimated by DLS. Moreover the size distribution of the lateral size of the flakes was obtained from three different AFM pictures; the images were analyzed using ImageJ Fiji Software (Figure 1B). Experimental FTIR spectra for GO and GO/CU showing the presence of the CU on the surface are reported (Figure 2). In Figure 2A, the spectrum of GO shows IR absorption bands corresponding to specific chemical functionalities: the 3400-cm^−1^-wide band can be linked to the O-H stretching vibration, the band at 2923 cm^−1^ is ascribed to the C–H bond vibration, the 1731 cm^−1^ peak corresponds to C=O stretching, the peak at 1624 cm^−1^ is ascribed to C=C stretching, the 1394 cm^−1^ signal corresponds to OH deformation, and the bands at 1235, 1069, and 987 cm^−1^ are characteristic of functional groups containing oxygen.

In Figure 2B, the GO/CU spectrum displays a strong peak at 800 cm^−1^ that indicates C–H stretching vibrations in curcumin [48]. Another peak is visible at 1020 cm^−1^ and is associated with curcumin superior C–O–C stretching vibrations [49]. In addition, 1270 cm^−1^ and 1560 cm^−1^ peaks are present in the spectrum, which are linked to the C–O vibrations and C=N vibrations of curcumin [50]. The peak at 1603 cm^−1^ is associated with the phenolic group stretching of curcumin, and the peak at 1730 cm^−1^ indicates the amide I band stretching [51]. Finally, the peak at 3000 cm^−1^ indicates the presence of asymmetric and symmetric stretches typical of graphene shown in Figure 2B [52].

### 2.2. Effect of GO and GO/CU on C. parapsilosis Planktonic Cells

#### 2.2.1. Growth Curve

**Figure 3:**
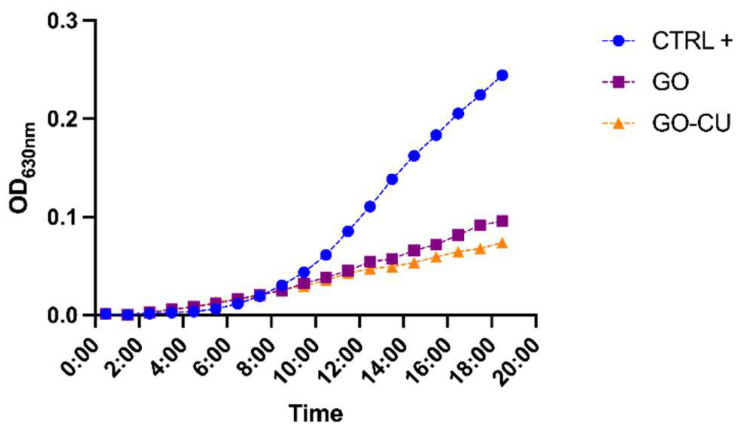
Growth curve of *C. parapsilosis* incubated with plastic (CTRL), GO, and GO/CU. O.D_630_ was monitored every hour for 18 h.

The effects of the two surfaces on planktonic *C. parapsilosis* cells were initially determined by growth curve evaluation [53,54]. A *C. parapsilosis* suspension at a final concentration equal to 10^4^ CFU/mL was added to GO, GO/CU, and plastic surfaces, and OD 630 nm was monitored every 60 min for 18 h. Both of the functionalized surfaces inhibited the growth of the *Candida* cells, showing a very low OD increase in comparison with cells incubated on plastic surfaces (Figure 3). The GO/CU surface appeared to benefit from the antimicrobial synergy between GO and curcumin, showing an even larger viability inhibition than the GO surface, which was perceptible after 14 h of incubation.

In the study conducted by Elbasuney S. and El-Sayyad G., the kinetic growth curve of *Staphylococcus aureus* on silver nanoparticle-coated medical cotton fibers was obtained. Similarly, to what we performed with GO and GO/CU on yeast, the authors evaluated the inhibition of bacterial growth of the antimicrobial material (silver-loaded) compared to the control (cotton fiber only). The absorbance of the microbial growth was evaluated at 2 h time intervals for 24 h. The results of the growth kinetics demonstrated a strong inhibition of microbial growth by the cotton functionalized with silver nanoparticles compared with the cotton fibers alone [54].

#### 2.2.2. Reactive Oxygen Species Assay

**Figure 4:**
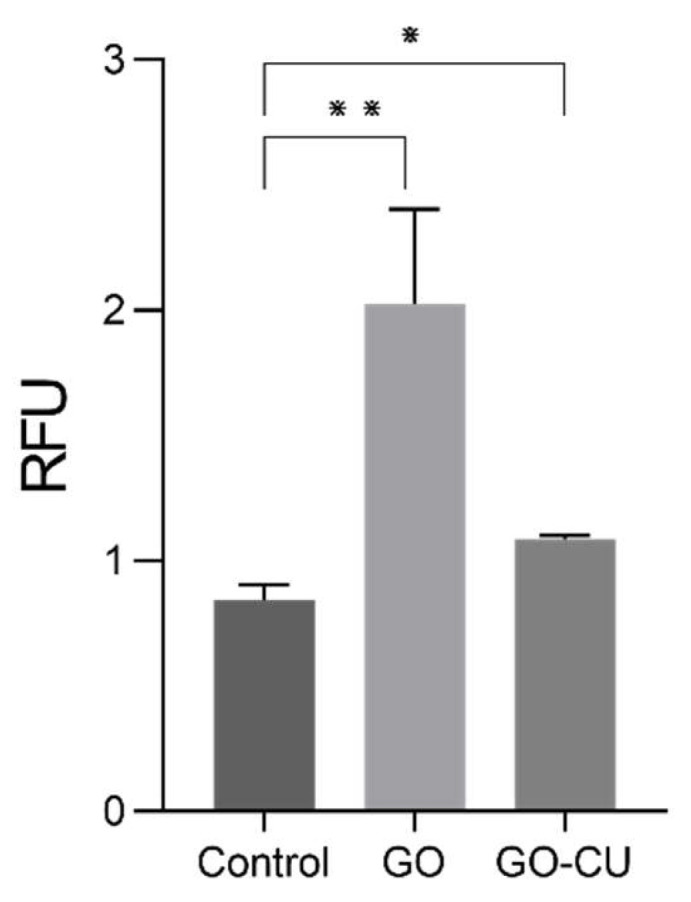
Intracellular ROS generation of *C. parapsilosis* cells in contact with plastic (control) surfaces, GO, and GO/CU after 6 h of incubation. Standard deviations of three independent experiments are represented by error bars. *p* values < 0.05 were considered significant: * < 0.05, ** < 0.005. RFU: relative fluorescent unit.

To explore the early damage caused by the GO and GO/CU surfaces to *C. parapsilosis*, we measured intracellular ROS generation through the DCFDA. *Candida* cells were put in contact with 96-well plates covered by GO and GO/CU and without coating (control) for 6 h and then, the ROS production was measured. The GO-coated surface induced strong production of intracellular ROS compared to the plastic surface, as already reported in the literature [20,22], while GO/CU also induced ROS production but to a lesser extent compared to GO (Figure 4). This result suggests that the GO/CU surface produced less early oxidative stress damage than GO in yeast eukaryotic cells but still showed a statistically significant (*p*-value < 0.05) difference compared to the plastic surface control. The induction of ROS production has been documented for bacteria in different studies [41,55], while for *Candida* species, even though the antifungal effect of curcumin has been reported [56], only one paper reported the production of ROS induced by curcumin for *C. albicans* [57]. The reduced oxidative stress in yeast cells can be a possible explanation of the attenuated cytotoxicity of GO/CU compared to GO, according to previous studies [20,22,31].

### 2.3. Effect of GO and GO/CU Surfaces on C. parapsilosis Early Adhesion and Biofilm Formation

#### 2.3.1. *C. parapsilosis* Coated Surface Adhesion

**Figure 5:**
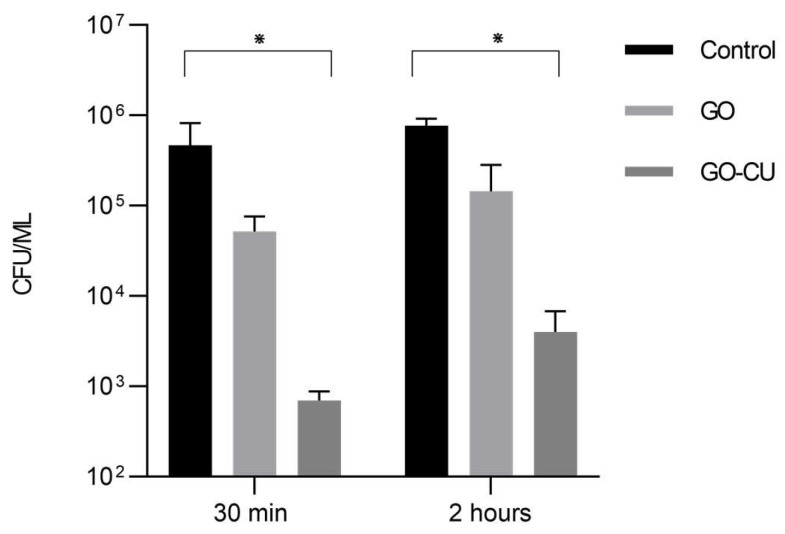
Adhesion assay. *Candida* cells were incubated with plastic, GO, and GO/CU for 30 min and two hours. Adherent cells were collected and enumerated by plating serial dilutions on Sabouraud agar plates and counting the CFU. Standard deviations of three independent experiments are represented by error bars. *p* values < 0.05 were considered significant: * < 0.05.

The adhesion of microbial cells to a biotic or abiotic surface is the first step toward the formation of a biofilm [5]; we therefore analyzed the ability of the GO- and GO/CU-coated surfaces to inhibit *C. parapsilosis* adhesion. Microbial cells were put in contact with GO, GO/CU, and uncoated polyurethane disks for 30 min and two hours, and then non-adherent cells were washed away and the CFU were counted. As shown in Figure 5, the GO/CU surfaces were able to prevent *C. parapsilosis* adhesion, showing a reduction in the number of viable cells attached to the surface at 30 min and at two hours while the GO surface did not show statistically significant differences compared to the control plastic disk. In our previous study [38], we demonstrated that the GO/CU surface was able to inhibit the adhesion of *C. albicans*. Most of the work presented in the literature reported the efficacy of the graphene scaffold linked to various antimicrobial substances against bacteria. As far as we know, this is the first work concerning the effect of nano-structured surfaces against the emerging pathogen *C. parapsilosis*.

#### 2.3.2. Crystal Violet Assay

**Figure 6:**
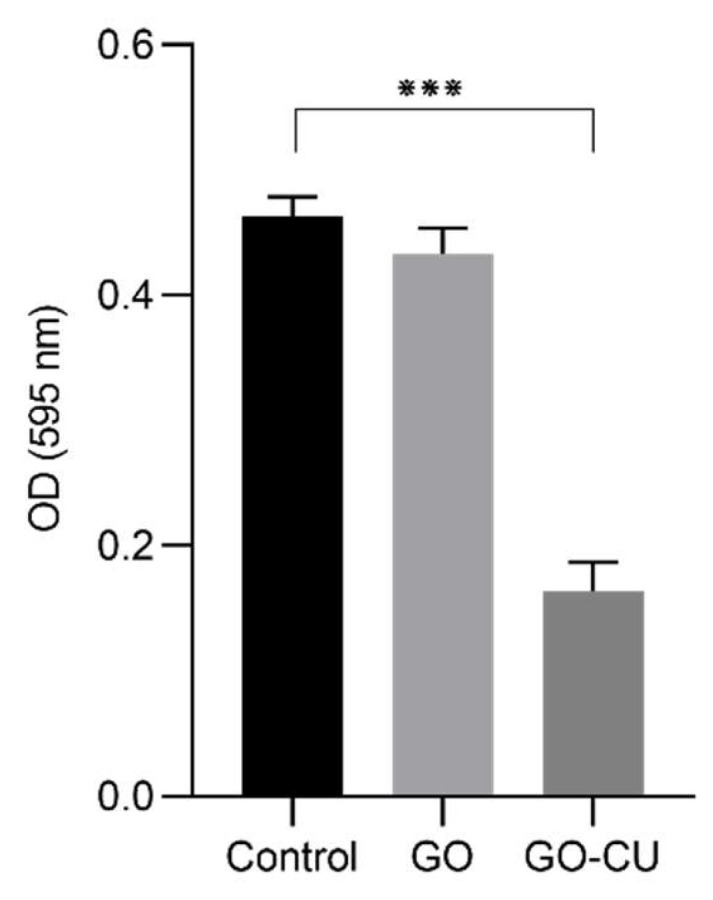
CV quantification of biofilm formation after 24 h on plastic, GO, and GO/CU. Biofilm biomass was stained with crystal violet, and then absorbance was read at 595 nm. Standard deviations of three independent experiments are represented by error bars. *p* values < 0.05 were considered significant: *** < 0.001.

We analyzed whether the coated surfaces were able to inhibit early-stage biofilm formation. *C. parapsilosis* cells were incubated for 24 h in contact with coated and uncoated surfaces, after which all non-adherent cells were washed away, and early-stage biofilm was stained with crystal violet (CV) [58]. Figure 6 shows the CV staining optical density (OD) of the three different surfaces. No differences between the GO surfaces and plastic disks (control) were detected, while the GO/CU surfaces inhibited the formation of *C. parapsilosis* biofilm, showing a decrease in biofilm biomass (*p* < 0.0001). The antibiofilm effect of GO has been documented for bacteria [59,60] but, as already described for *C. albicans* [38], no anti-adhesive or anti-biofilm properties have been detected for GO, while the conjugation with CU strongly enhances the anti-biofilm activity [38].

#### 2.3.3. Live and Dead Assay

**Figure 7:**
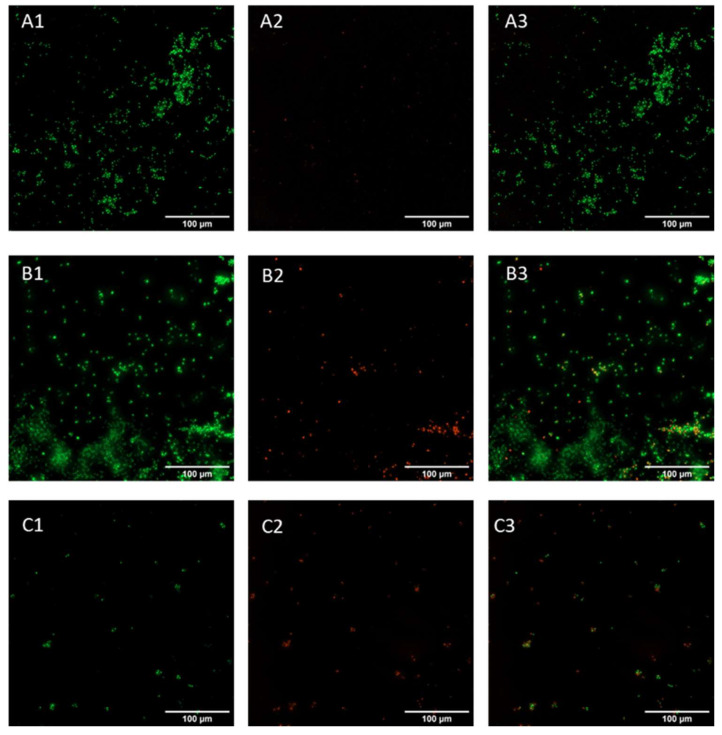
Live and dead representative fluorescent images of *Candida* biofilm grown on a plastic surface (**A**) and GO (**B**) and GO/CU (**C**) surfaces for 24 h. Live cells are stained green (**A1**–**C1**), and dead cells are stained red (**A2**–**C2**). Images were merged (**A3**–**C3**) with ImageJ Fiji software. Images were taken using the Cytation 5 Cell Imaging Multi-Mode Reader (Agilent, Santa Clara, CA, USA). The scale bar is 100 µm.

Live and dead assay was conducted on early-phase biofilm (24 h incubation) to confirm the data obtained with crystal violet staining. This assay allows the visualization of viable cells, stained green, and dead cells, stained red. *Candida* cells appeared viable in the control sample (Figure 7A), with no red cells present on the surface; a similar morphology was displayed in the GO samples (Figure 7B), while cells incubated with GO/CU appeared to be less viable compared to those subjected to plastic and GO (Figure 7C), with a higher proportion of red cells, indicating cell membrane perturbation and subsequent cell death.

### 2.4. Effect of GO and GO/CU Surfaces on C. parapsilosis Mature Biofilm Formation

#### 2.4.1. Fluorescein Diacetate Assay (FDA) 

**Figure 8:**
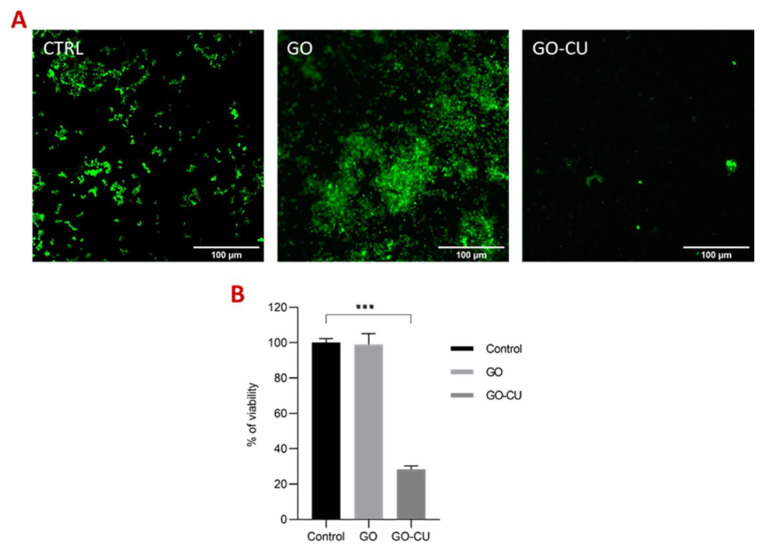
Fluorescein diacetate assay (FDA). (**A**) Representative confocal microscopy images of *Candida* biofilm grown for 72 h on plastic (CTRL), GO, and GO/CU surfaces. Images were taken at 40X magnification. The scale bar is 100 μM. (**B**). The viability (percentage of control) of biofilm-associated cells was evaluated using FDA. Fluorescent intensities were determined by ImageJ Fiji software. Standard deviations of three independent experiments are represented by error bars. *p* values < 0.05 were considered significant: *** < 0.001.

FDA was conducted after 72 h of incubation to evaluate the viability of the cells in a mature biofilm formed on the three different surfaces. FDA allows the quantification of viable cells thanks to their ability to convert the molecule FDA into fluorescent fluorescein by non-specific intra- and extracellular esterases [58]. Figure 8A represents *C. parapsilosis*-viable biofilm-associated cells, visualized by confocal microscopy in contact with uncoated surfaces, the GO-coated surface, and the GO/CU-coated surface. The analysis showed a 72% reduction of viability of biofilm-associated cells grown on GO/CU surfaces (Figure 8B) compared to plastic disks and GO-coated disks.

#### 2.4.2. EPS Staining

**Figure 9:**
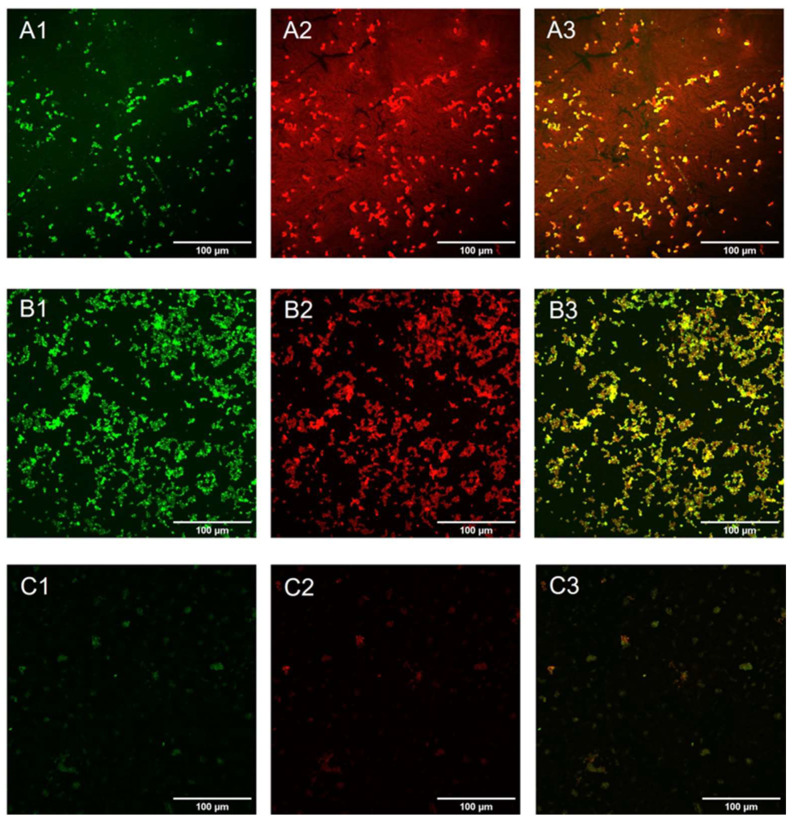
Confocal microscopy representative images of *Candida* biofilm cells grown on plastic (**A**), GO (**B**), and GO/CU (**C**) for 72 h. Cells were stained with Syto 9 (**A1**–**C1**) and ConcA (**A2**–**C2**). Images were merged (**A3**–**C3**) with ImageJ Fiji software. The scale bar is 100 μM.

**Figure 10:**
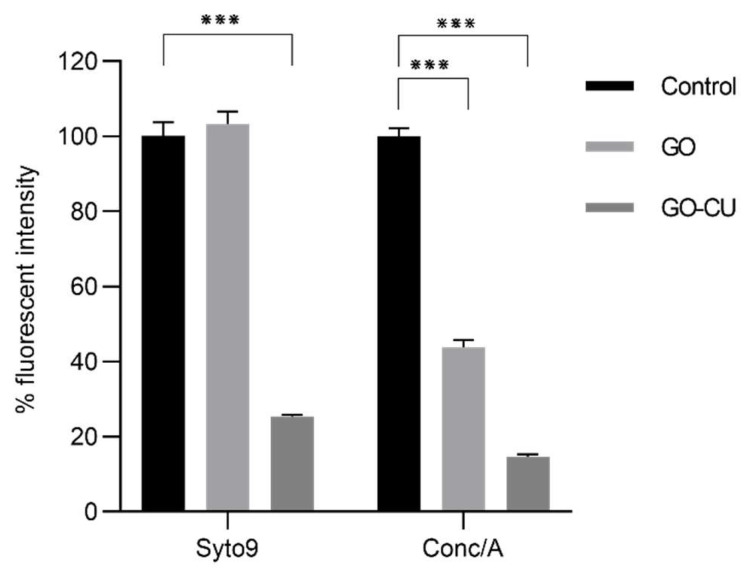
Fluorescence intensity analysis of Syto9/ConcA (percentage of the control) on the three different surfaces, plastic (control), GO, and GO/CU. Fluorescent intensity was determined by ImageJ Fiji software. Standard deviations of three independent experiments are represented by error bars. *p* values < 0.05 were considered significant: *** < 0.001.

To investigate how the coated surfaces were able to prevent the formation of the mature biofilm composed of cells embedded in extra-polymeric substances (EPS), *C. parapsilosis* cells were incubated with plastic disks and GO- and GO/CU-coated surfaces for 72 h; then, the biofilms were stained with Syto9 and Concanavalin A-Texas Red conjugate and visualized using confocal microscopy [61]. Concanavalin A selectively binds to polysaccharides, including alpha-mannopyranosyl and alpha-glucopyranosyl residues, present in the candida cell wall and EPS. Figure 9 shows the *C. parapsilosis* biofilm on uncoated surfaces (A), GO (B), and GO/CU (C). For each surface, Syto9 (1), Concanavalin-A (2), and merged images of both staining (3) are shown. In the control sample, cells look viable and organized in the typical architecture of the mature biofilm, with numerous cells surrounded by EPS and numerous biofilm-associated cells on GO-coated surfaces, confirming the data obtained from FDA analysis, but EPS are poorly present and less homogenous than in the control. As expected, there were few cells on the GO/CU-coated surface, and they were unable to produce EPS. Figure 10 shows the analysis of fluorescent intensity; while there are no differences in the number of cells on the GO surfaces compared to plastic disks, the production of EPS seems to be inhibited in GO-coated disks compared to the control. To date, the effect of GO on the synthesis of EPS after long-term incubation has not been investigated for *Candida* species. Ramasamy and collaborators [62] investigated the effect of GO alone and conjugated with alizarin (AZ) against *C. albicans* biofilm. They quantified the biomass production by crystal violet assay after 24 h of incubation, revealing no differences between GO and the control sample; on the other hand, they measured the hyphal production, revealing an unpaired/delayed hyphal production, speculating that GO may impact the cell separation process. Our data showed an anti-microbial activity of GO against the non-adherent yeast cells, stimulating ROS production and inhibiting cell replication. It is possible that the prolonged incubation could favor a better contact between the yeast and the GO surface, allowing the latter to elicit anti-microbial mechanisms, causing an alteration in *C. parapsilosis* metabolism that leads to an inhibition of the secretion of EPS, but further investigations are needed to clarify this aspect.

## 3. Discussion

*Candida* spp. are the fifth leading cause of bloodstream infections (BSI) worldwide. Although *C. albicans* remains mainly associated with such infections, recent epidemiological findings show increasing incidence of other *Candida* spp. and in particular, *C*. *parapsilosis*. The main issue is linked to the strong ability of *C. parapsilosis* to form biofilms on medical devices, which allows the yeast to diffuse into the bloodstream. Nowadays, the absence of clinically approved anti-biofilm drugs results in the removal of the infected devices when biofilm-related infection is suspected. However, this is not always feasible, and antimicrobial treatment may be necessary with high concentrations of antifungals for lock therapy in combination with systemic treatment. Unfortunately, these therapies are often ineffective and contribute to the onset of antifungal resistance. Thus, there is a clear need for effective preventative measures, such as thin coatings that can be applied to medical devices to prevent the attachment, colonization, and formation of device-associated biofilms. The crucial aspect in preventing biofilm formation concerns the early stages of pathogen adhesion, when the interaction with the abiotic surface is still reversible but becomes irreversible when the biofilm is formed. Accordingly, research has been focused on preventing the initial attachment of microbial cells onto the surfaces of materials used for indwelling biomedical devices. In the last few decades, an increasing number of researchers have begun to coat various materials such as those used for medical implants to prevent infection [22,63]. It is now known that GO has broad-spectrum antimicrobial activity, both when used in solution and when deposited or coated on surfaces [25]. Curcumin is the principal curcuminoid of the turmeric plant *Curcuma longa*. Similarly, the antiviral, antifungal, and antibacterial properties of curcumin have long been known together with proven anti-biofilm properties [34,38,64]. Rai and co-workers reported the antimicrobial mechanism of action of curcumin linked to the interference of cell division by targeting the filamenting temperature-sensitive mutant Z (FtsZ), relevant in the early stages of cell division, as it is responsible for the formation of the septum that promotes the division of the bacterial cell [29]. Although the binding site of curcumin in FtsZ is not known, other studies report the ability of curcumin to prevent the assembly of the cytoskeletal protein FtsZ. The authors of this study evaluated the intracellular entry of fluorescent probes such as propidium iodide and calcein following treatment with different concentrations of curcumin of Gram+ and Gram− bacteria [65]. Bellio P. and collaborators highlighted the ability of curcumin to inhibit the SOS response in *Escherichia coli* induced by levofloxacin [66]. In this work, GO and curcumin were combined in a green formulation for surface coating to prevent the adhesion and biofilm formation of *C. parapsilosis*.

GO can act as a scaffold and spontaneously interact with curcumin, which, with its known chemical instability and water insolubility, is easily captured and stabilized by GO sheets. The CU is capable of interfering with *C. parapsilosis* adhesion to surfaces and biofilm formation. Furthermore, the synthesis of GO/CU was carried out with the same procedure previously described by our group [30], where the cytotoxicity assays showed excellent safety results on human fibroblast cell lines, also avoiding the hemolysis of red blood cells.

The FTIR characterization of the GO and GO/CU-coated surfaces clearly showed the curcumin attached to the surface.

In work by Alalwan H. et al. [64], curcumin was able to interfere with the expression of biofilm-associated genes, such as ALS1 and ALS3 adhesins of *C. albicans*, demonstrating a specific ability to interfere with the early stages of fungal adhesion. The results obtained from the early-stage adhesion tests of *C. parapsilosis* at 30 min and 2 h show how the GO/CU surface can significantly inhibit fungal early adhesion compared to uncoated or GO-only-coated polyurethan disks. Furthermore, results obtained from FDA and Syto9/Concanavalin A-Texas Red conjugate staining demonstrate the strong anti-biofilm properties of the GO/CU-functionalized surface compared to controls. The use of dyes able to specifically bind different classes of macromolecules present in the biofilm matrix clearly show how the few yeasts adhering to the GO/CU surface are isolated planktonic cells without any three-dimensional organization and the inability to produce EPS. Overall, GO/CU-functionalized surfaces hold great promise for biomedical device applications.

## 4. Materials and Methods

### 4.1. GO and GO/CU Surface Preparation

A total of 100 μL of 1 mg/mL GO suspension (GrapheneA, Cambridge, MA, USA) was deposited on sterile 10 mm plastic coverslips (Thermo Fisher Scientific, Waltham, MA, USA) to obtain a uniform GO substrate. For GO/CU samples, 50 μL of ethanol-dissolved CU (Sigma–Aldrich, Milan, Italy) at a concentration of 2 mg/mL was deposited on the GO coatings.

### 4.2. Characterization of Samples

GO was characterized using a Zetasizer Nano ZS (Malvern, Herrenberg, Germany), and the lateral size was calculated as reported previously. Representative images were obtained with NanoWizard II AFM (JPK Instruments AG, Berlin, Germany), as reported previously. The chemical analysis of the surfaces was obtained using attenuated total reflectance-Fourier transform infrared (ATR-FTIR) spectroscopy (Spectrum One spectrometer from Perkin Elmer, Waltham, MA, USA) as previously reported [47]. The surfaces were directly laid on the ATR crystal, and the spectra were recorded in the wave number range of 4000–550 cm^−1^.

### 4.3. Clinical Strain

The *C. parapsilosis* strain was isolated from blood cultures and previously characterized as the HBF strain [38]. *Candida* cells were grown in RPMI (Thermo Fisher Scientific, Waltham, MA, USA) medium at 30 °C overnight on 150 orbital shakers. Yeast cells were grown on BCG agar (*Candida* bromcresol green) for 48 h at 30 °C.

### 4.4. Growth Curve

The growth curve is a spectrophotometric investigation in which the increasing optical density (OD) of microorganisms in culture media is compared to their proliferation. The elective wavelengths used to monitor optical density range from 600 to 630 nm [67]. To evaluate the differential growth inhibition of the GO- and GO/CU-coated surfaces on *C. parapsilosis*, a suspension of approximately 10^4^ CFU/mL in Muller Hinton Broth (Sigma–Aldrich; Sant Louis, MO, USA) was prepared and added to 96-well GO- and GO/CU-coated plates and non-coated flat-bottom 96-well plates (Corning Incorporated, Corning, NY, USA) as a control. To remove the background signal of GO and GO/CU, a blank subtraction was necessary. Triplicate samples of GO- and GO/CU-coated wells were incubated with Muller Hinton Broth alone. The mean OD value was then used as the blank and was subtracted from the OD value obtained from samples incubated with GO and GO/CU. The plates were then incubated in a Cytation 5 Cell Imaging Multi-Mode Reader (Agilent, USA) with a kinetic protocol of 18 h monitoring of OD (630 nm) changes every 60 min. The incubation was performed at 37 °C, 5% CO_2_, on an orbital continuous shaker at 250 rpm. The optical density was measured at ʎ = 630 nm as described before.

### 4.5. ROS Assay

ROS production was detected using the DCFDA/H2DCFDA—Cellular ROS Assay Kit (Abcam, Cambridge, United Kingdom) following a previously published protocol [68,69] Briefly, *C. parapsilosis* cells were grown overnight in RPMI medium, then, cells were sub-inoculated 1:100 in fresh RPMI medium and grown at 37°C to an OD (630 nm) corresponding to 0.6. Then, cells were washed once in PBS medium, resuspended in fresh medium, and 100 µL of the yeast suspension was added to the plastic, GO- and GO/CU-coated surfaces and incubated at 37 °C for 6 h. Then, the yeast suspension was transferred to a 1.5 mL Eppendorf, washed once, and then incubated with 10 μM DCFDA in PBS for 30 min at 37 °C according to manufacturer’s instruction. Cells were then washed twice in PBS, after which 50 µL of the bacterial suspension was transferred to 96-well plates for fluorescence (Life TECHNOL-OGIES, Carlsbad, CA, USA) measurement using a Cytation 5 Cell Imaging Multi-Mode Reader (Agilent, USA) with Ex/Em = 485/535 nm. All assays were performed in triplicate.

### 4.6. Adhesion Assay

*C. parapsilosis* cells, grown overnight as previously described, were diluted to 0.5 McFarland, corresponding to about 5 × 10^6^ CFU/mL in fresh RPMI medium supplemented with 0.25% glucose. One mL of the diluted cells was added to 24-well plates (Thermo Fisher Scientific, USA) containing plastic disks (control), GO-coated disks, and GO/CU-coated disks for 30 min and 2 h. At each time point, disks were washed with sterile phosphate-buffered saline (PBS) (Sigma–Aldrich, Gillingham, UK) to remove all non-adherent cells, and disks were sonicated for 10 min at 35 KHz (Ultrasonic bath, Fisher scientific, Loughborough, UK) and vortexed for 30 **s**. Serial dilutions of adherent *C. parapsilosis* cells were plated on BCG agar, and CFU were counted after 48 h of incubation. Each essay was conducted in triplicate and repeated twice.

### 4.7. Crystal Violet Biofilm Quantification Assay

Cristal violet biofilm quantification assay was performed as previously described [58]. Yeast cells were grown overnight as previously described. The inoculum was then adjusted to 0.5 McFarland in RPMI, and 1 mL of the suspension was added to a 24-well plate (Thermo Fisher Scientific, USA) containing plastic disks and GO- and GO/CU-coated disks. The plates were incubated at 37 °C for 24 h, and then all non-adherent cells were carefully washed away by rinsing the wells three times with PBS. Subsequently, 500 µL of crystal violet stain (Sigma–Aldrich, UK) was added to each well and incubated at room temperature for 45 min. Then, the stain was removed, each well was washed three times by PBS, and allowed to dry. Then, 100 µL of ethanol 100% was added to dissolve the crystal violet, the solution was transferred to a 96-well plate, and the absorbance was read at 560 nm. Each assay was conducted in triplicate and repeated twice.

### 4.8. LIVE and DEAD Assay

Yeast cells were prepared as described above. After 24 h incubation, cells were stained using the LIVE/DEAD™ BacLight™ Bacterial Viability Kit (Thermofisher Scientific, USA) according to manufacturer’s instructions. Samples were visualized using the Cytation 5 Cell Imaging Multi-Mode Reader (Agilent, USA) with excitation wavelengths of 469 nm and 586 nm and emission at 525 nm and 647 nm for green and red channels, respectively.

### 4.9. FDA 

FDA was performed as already described [58]. Microbial cells, grown overnight in RPMI, were diluted to 0.5 McFarland in fresh medium. One mL of the suspension was added to 24-well plates containing plastic disks and GO- and GO/CU-coated disks and incubated 72 h at 37 °C. Then, the disks were washed three times with PBS. Fluorescein diacetate (FDA) (Thermo Fisher Scientific, MA, USA) at a concentration of 1 mg/mL was dissolved in acetone to prepare a stock solution, stored at −20 °C, and then added to each well at a final concentration of 10 µg/mL and incubated at 37 °C for 1 h in the dark. The fluorescence of viable stained cells was visualized by confocal microcopy (Eclipse Ti-E Nikon, Nikon Europe B.V, Amstelveen, The Netherlands) with excitation at 480 nm and emission at 525 nm, and then the images were analyzed using ImageJ Fiji Software (Version 1.53 c).

### 4.10. Biofilm Confocal Microscopy

*C. parapsilosis* biofilm was prepared as described above. After 72 h of incubation, disks were carefully washed, and the biofilm was stained with Syto9 (Thermo Fisher Scientific, MA, USA) at a final concentration of 250 nM and with Concanavalin A-Texas Red conjugate (Thermo Fisher Scientific, MA, USA) at a final concentration of 100 µg/mL and incubated in the dark for 1 h at room temperature [61]. Disks were then washed with distilled water to remove any excess stain and were visualized using confocal microscopy with excitation at 480 nm and emission at 525 nm for the green channel and excitation at 595 nm and emission at 620 nm for the red channel. Images were analyzed using ImageJ Fiji Software (Version 1.53 c).

### 4.11. Statistical Analysis

All statistical analysis were performed using GraphPad Prism version 9.0.0 for Windows, GraphPad Software (San Diego, CA, USA). Analyses were done using analysis of variance (one-way ANOVA), and *p* values < 0.05 were considered significant.

## 5. Conclusions

GO possesses antimicrobial properties already studied and can be easily functionalized thanks to the reactive chemical groups and the high surface area. Curcumin is also a substance with antimicrobial action but with poor solubility and bioavailability. We demonstrate that the union of the two composites leads to the formation of a stable and effective nanomaterial useful to prevent fungal adhesion and biofilm formation. Moreover, for the first time, the antimicrobial properties of GO/CU have been tested against the emerging pathogen *C. parapsilosis* associated with catheter-related bloodstream infections. The next step will concern the functionalization with GO/CU of a plastic catheter to carry out fluidics’ studies. This will allow us to reliably evaluate the stability and durability of the nanomaterial and then move on to an in vivo application with animal models. Considering our preliminary data, we believe that this nanomaterial has the potential to be used in the field of medical device functionalization.

## Data Availability

Data is contained within the article.
